# Characterizing Social Determinants of Health in Patients With Type 2 Diabetes and Liver Disease: Cross-Sectional Survey Study

**DOI:** 10.2196/91608

**Published:** 2026-06-15

**Authors:** Parnnate Wongsirisakul, Vincent Lingzhi Chen, Jonathan Troost, Ponni Perumalswami

**Affiliations:** 1 Division of Gastroenterology and Hepatology, Michigan Medicine, University of Michigan, 1500 E Medical Center Dr., Ann Arbor, MI, United States, 1 734-647-9252; 2 Michigan Institute for Clinical and Health Research, University of Michigan, Ann Arbor, MI, United States

**Keywords:** social determinants of health, type 2 diabetes, liver disease, survey, care delivery

## Abstract

**Background:**

The mortality rate from liver disease among people with type 2 diabetes mellitus (T2DM) increased by 20% between 2001 and 2018. There are marked racial and ethnic differences among people with T2DM at risk of metabolic dysfunction–associated steatotic liver disease (MASLD) and related complications.

**Objective:**

We aimed to investigate the distribution of individual-level social determinants of health (SDOH) in people living with both T2DM and MASLD.

**Methods:**

In this small cross-sectional study, patients (N=50) were recruited from a tertiary care general hepatology clinic to complete a survey that assessed potential determinants of health. We sought to oversample Black and Hispanic patients to better understand the prevalence of SDOH. Electronic health records were reviewed to determine stage of liver disease, and these data were linked to survey results to identify the distribution of individual-level determinants of health in patients with cirrhosis.

**Results:**

Black and Hispanic respondents were more likely to report more experiences of racial discrimination, worries about being discriminated against, and group-based medical mistrust, especially regarding unsupportive health care providers. Cirrhosis groups tended to have lower incomes and less coverage from private health insurance. However, no substantial trends were observed in the distribution of health literacy, discrimination, and diabetes stigma among patients with and without cirrhosis.

**Conclusions:**

These findings will inform a future study aimed at assessing and developing interventions to address the combined impact of individual- and neighborhood-level SDOH on health-related outcomes in patients with T2DM and MASLD.

## Introduction

Metabolic dysfunction–associated steatotic liver disease (MASLD) is a public health priority, as it affects 60% to 80% of people with type 2 diabetes mellitus (T2DM) and 100 million people in the United States [1,2]. MASLD comprises a spectrum of disease ranging from its early stages (largely asymptomatic simple steatosis) to cirrhosis, which can lead to life-threatening complications such as ascites, variceal bleeding, and liver cancer [3]. Complications from MASLD disproportionately affect people with T2DM. More than 5% of patients with T2DM who are above the age of 50 years have cirrhosis (vs approximately 1% in the general population) [1,4], and the mortality rate from liver disease among people with T2DM increased by 20% between 2001 and 2018 [3]. There are marked racial and ethnic differences among people with T2DM at risk of MASLD and related complications. In the United States, MASLD is most common among Hispanic individuals, followed by White and then Black individuals [5].

Social determinants of health (SDOH) contribute to disparities in both disease prevalence and health outcomes among people living with both T2DM and MASLD. Individual-level factors associated with risk of complications include food insecurity, lower income, and lower educational level [6]. However, there are limited data on neighborhood-level SDOH and how location impacts health additively or synergistically with individual- or household-level factors. This has restricted our understanding of disparities in MASLD or mechanisms through which they may result in liver-related complications, which may require different interventions to reduce these disparities and improve health outcomes. In our prior work, we evaluated the impact of neighborhood-level SDOH in a diverse Michigan Medicine cohort of 18,915 adults with MASLD, of whom 50% had T2DM. Higher disadvantage scores were associated with higher mortality and risk of cardiovascular disease, whereas higher advantage scores were linked to lower mortality, cardiovascular disease risk, and rate of hepatic decompensation [7]. These findings provide preliminary data on the fact that neighborhood-level SDOH are associated with clinically relevant outcomes in our patients with MASLD. Considering our prior research on neighborhood-level SDOH, in this study, we aimed to investigate the distribution of individual-level SDOH in patients with T2DM and MASLD.

## Methods

### Study Sample and Data Source

We evaluated SDOH in a small cohort of patients with T2DM and MASLD using data from electronic health records (EHRs) and a survey with measures selected using a health disparities conceptual model adapted from Warnecke et al [8,9]. Michigan Medicine is a tertiary care center that services patients from rural, suburban, and urban areas throughout the state. The hepatology clinics offer specialized care and programs in cirrhosis, steatotic liver disease, and liver transplantation. Patients (N=50) scheduled for in-person visits were recruited from the Michigan Medicine general hepatology clinic between September 1, 2024, and May 1, 2025. Eligible participants were aged 18 years or older, had a diagnosis of T2DM based on established criteria [10], were fluent in English, and had visited the Michigan Medicine hepatology clinic at least once in the previous 2 years (January 1, 2021, to December 31, 2022). We sought to oversample Black and Hispanic patients to better understand the prevalence of SDOH in diverse populations, particularly those with higher rates of determinants of health. From 2019 to 2024, a total of 775 Hispanic patients (4% of the total hepatology clinic pool) and 1274 Black patients (7%) were seen. This would be equivalent to recruiting roughly 2 Hispanic and 4 Black patients for a sample size of 50. To oversample, we aimed to double this number and recruit 4 Hispanic and 7 to 8 Black patients. We followed the STROBE (Strengthening the Reporting of Observational Studies in Epidemiology) reporting guidance [11].

This was a cross-sectional study design that combined a one-time survey with data from the EHR. The survey was administered 1:1 by a trained study coordinator and lasted approximately 30 minutes. Consecutive patients were recruited from upcoming appointments until target numbers for racial and ethnic categories were met. A total of 114 patients were eligible to participate in this study, but 3 (2.6%) exceeded target patient population numbers, so 111 (97.4%) patients were approached. Of these 111 patients, 40 (36%) were unable to be reached, and 21 (18.9%) refused to participate, stating reasons such as lack of interest and scheduling conflicts. Thus, 50 patients consented and completed the survey. Medical records for all individuals who completed the survey were reviewed by a trained study coordinator. Hepatology care provider notes were reviewed for a diagnosis of cirrhosis [12]. Laboratory tests, comorbidities, diabetes medications, and race and ethnicity were also extracted from the EHR. Each EHR variable and survey instrument was completed without any missing data points.

### Survey Design and Measures

**Figure 1.**
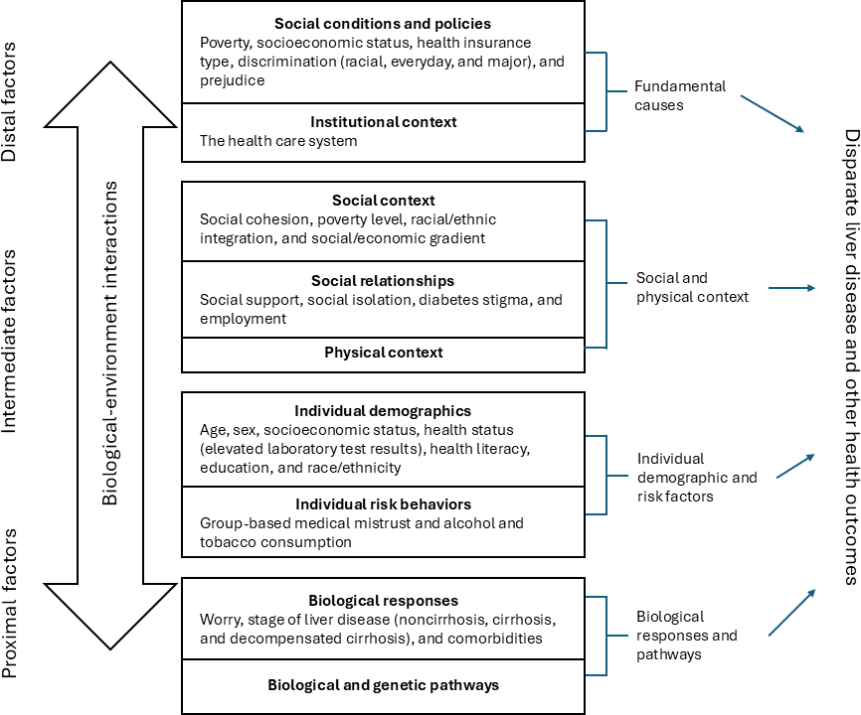
Health disparities conceptual model by Warnecke et al [8,9] adapted for type 2 diabetes mellitus and metabolic dysfunction–associated steatotic liver disease patient-reported individual-level social determinants of health.

The health disparities conceptual model by Warnecke et al [8,9] (see Figure 1 [8,9] for study adaptation) was chosen for this study as it combines the role that SDOH and other biological factors play in contributing to health burden and outcomes. It has been successfully used to investigate disparities in other diseases such as liver, breast, and colorectal cancers [13]. This conceptual model by Warnecke et al [8,9] also aligns with the National Institute of Minority Health and Health Disparities research framework—the latter of which reflects an evolving conceptualization of factors relevant to the understanding and promotion of minority health and to the understanding of and reduction in health disparities [14]. In the model by Warnecke et al [8,9], “distal” factors based on social conditions, policies, and institutions; “intermediate” factors such as social and physical context and individual demographics; and “proximal” factors such as individual behaviors and biological responses all contribute to disparities in health outcomes. The surveys administered encompassed the SDOH domains listed in Multimedia Appendix 1. Questions related to health literacy, health-related stigma, alcohol intake, and medical mistrust were derived from validated scales explained below. Income, educational level, health insurance, English-language proficiency, and tobacco and injection drug use were single-item self-reported measures obtained from the survey.

Health literacy was assessed through the Rapid Estimate of Adult Literacy in Medicine–Short Form [15]—a test composed of 7 commonly used health terms that the participant is asked to read aloud. A total of 0 correct words correlates to a literacy level of third grade and below, 1 to 3 correct words correlate to a fourth- to sixth-grade level, 4 to 6 correct words correlate to a seventh- to eighth-grade level, and 7 indicates at least a high school–level literacy and the ability to read most patient materials. Variables such as diabetes stigma and group-based medical mistrust were scored on a 5-point Likert scale (1=“strongly disagree”; 5=“strongly agree”). The group-based medical mistrust score was divided into 3 sections: “suspicion” (answer range 6-30), “group disparities in health care” (range 3-15), and “lack of support from health care providers” (range 3-15) [16]. Because the group disparity statements are phrased positively (ie, “in most hospitals, people of different ethnic groups receive the same kind of care”), a higher score means less disparities. Meanwhile, the diabetes stigma score was divided into “treated differently,” “blame and judgment,” and “self-stigma” domains [17]. Each section of the diabetes stigma score ranges from 6 to 30 except for “blame and judgment,” which ranges from 7 to 35. The racial discrimination and major discrimination scores range from 0 to 9, worry ranges from 0 to 12, and the Everyday Discrimination Scale ranges from 0 to 30.

### Ethical Considerations

All research activities were approved and reviewed by the University of Michigan Institutional Review Board prior to study initiation under approval HUM00243618. Written informed consent was obtained for all participants.

### Statistical Analysis

Participants were grouped by race and ethnicity to identify any trends in disease progression and SDOH distribution. They were specifically divided into Black and Hispanic participants and non–Black and Hispanic participants because using grouping solely based on Black or Hispanic race and ethnicity would have rendered the sample sizes too small. To avoid misinterpretation from racial and ethnic aggregation, 2 additional analyses between Hispanic and non-Hispanic individuals were performed—one in which the Black participants were grouped with non-Hispanic respondents and one in which the Black participants were excluded from the comparison entirely (Multimedia Appendix 2). No significant differences were observed in the supplemental analyses when compared to the original grouping of Black and Hispanic participants vs non–Black and Hispanic participants.

The intended purpose of this study was formative hypothesis generation; thus, *P* values were descriptive and should be interpreted cautiously. The following analysis plan prespecifies discrimination and medical mistrust as primary SDOH constructs, with stigma and health literacy as secondary constructs. This prioritization resulted from the findings of our previous study on primary care physician–related determinants of T2DM pharmacoinequity, in which physicians identified discrimination and medical mistrust as key barriers to newer, more effective medication uptake in patients [18].

Answers to racial discrimination and major experiences of discrimination [19] questions were coded as 1 for “yes” and 0 for “no.” For worry questions developed by Krieger et al [20,21], the response options were o (“rarely” and “never”), 1 (“some of the time”), and 2 (“most of the time”). The expanded Everyday Discrimination Scale by Williams et al [19] followed a similar format, with responses of 0 (“never”), 1 (“once”), 2 (“two or three times”), and 3 (“four or more times”). The statistical tests for *P* values all used the nonparametric Kruskal-Wallis test for continuous variables and the Fisher exact test for categorical variables. For any continuous variables compared between 2 levels, the Hodges-Lehmann median differences were calculated.

## Results

### Sociodemographic Characteristics

**Figure 2.**
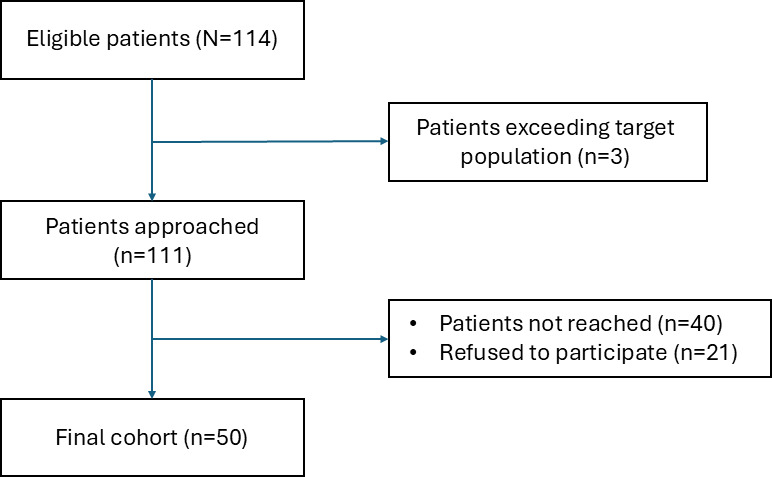
Patient flow diagram.

Figure 2 shows a flow diagram for recruitment and participation. The sociodemographic data are divided by race and ethnicity (Black and Hispanic participants vs not Black or Hispanic participants) in Table 1 and by stage of liver disease along with SDOH median scores in Table 2. Of the 50 survey participants, 7 (14%) were non-Hispanic Black or Hispanic individuals, and 43 (86%) were non-Hispanic White individuals. In comparison to White participants, Black and Hispanic participants were less often female (26/43, 60.5% vs 3/7, 42.9%, respectively), had a lower income, were more currently employed (14/43, 32.6% vs 3/7, 42.9%, respectively), and more commonly used Medicare or Medicaid. The average age and level of education were similar across races and ethnicities. A further breakdown of race and ethnicity and income is available in Multimedia Appendix 3.

**Table 1.** Sociodemographic characteristics divided by race and ethnicity^a^.

	Black and Hispanic participants (n=7)	Not Black or Hispanic participants (n=43)	*P* value
Demographics			
Age (y), mean (SD)	64 (8.7)	63 (9.1)	.80
Sex (female), n (%)	3 (42.9)	26 (60.5)	.38
Employed, n (%)	3 (42.9)	14 (32.6)	.59
Annual household income (US $), n (%)			.48
<35,000	3 (42.9)	9 (20.9)	
35,000-49,999	0 (0)	8 (18.6)	
50,000-74,999	2 (28.6)	5 (11.6)	
75,000-99,999	1 (14.3)	3 (7.0)	
>100,000	1 (14.3)	13 (30.2)	
Educational level, n (%)			.48
Lower than high school	0 (0)	1 (2.3)	
High school	0 (0)	9 (20.9)	
College	4 (57.1)	28 (65.1)	
Graduate degree	3 (42.3)	5 (11.6)	
Insurance type, n (%)			.69
Medicaid	2 (28.6)	7 (16.3)	
Medicare	3 (42.9)	14 (32.6)	
Marketplace (insurance purchased by the policyholder and not distributed through their employer)	0 (0)	2 (4.7)	
Private (employer)	2 (28.6)	20 (46.5)	

a Hodges-Lehmann median difference: 1.0 (IQR –6.0 to 9.0).

**Table 2.** Sociodemographic characteristics and social determinant of health (SDOH) median scores divided by stage of liver disease.

	No cirrhosis (n=10)	Compensated cirrhosis (n=28)	Decompensated cirrhosis (n=12)	*P* value
Demographics				
Age (y), mean (SD)	63.3 (7.65)	62.0 (9.32)	63.8 (9.74)	.84
Sex (female), n (%)	7 (70)	15 (53.6)	7 (58.3)	.66
Employed, n (%)	5 (50)	10 (35.7)	2 (16.7)	.25
Race and ethnicity, n (%)				.30
Black or African American	0 (0)	1 (3.6)	0 (0)	
Hispanic	2 (20)	4 (14.3)	0 (0)	
White, not Hispanic	8 (80)	21 (75)	12 (100)	
Other, not Hispanic	0 (0)	2 (7.1)	0 (0)	
Annual household income (US $), n (%)				.30
<35,000	0 (0)	10 (35.7)	2 (16.7)	
35,000-49,999	3 (30)	3 (10.7)	2 (16.7)	
50,000-74,999	1 (10)	4 (14.3)	2 (16.7)	
75,000-99,999	2 (20)	1 (3.6)	1 (8.3)	
>100,000	4 (40)	7 (25)	3 (25)	
Educational level, n (%)				.69
Lower than high school	0 (0)	1 (3.6)	0 (0)	
High school	2 (20)	4 (14.3)	3 (25)	
College	6 (60)	20 (71.4)	6 (50)	
Graduate degree	2 (20)	3 (10.7)	3 (25)	
Insurance type, n (%)				.04
Medicaid	1 (10)	8 (28.6)	0 (0)	
Medicare	3 (30)	10 (35.7)	4 (33.3)	
Marketplace	0 (0)	2 (7.1)	0 (0)	
Private (through employer)	6 (60)	8 (28.6)	8 (66.7)	
SDOH scores, median (IQR)				
Health literacy (0-7)	7.0 (7.0-7.0)	7.0 (7.0-7.0)	7.0 (7.0-7.0)	.07
Group-based medical mistrust				
Suspicion (6-30)	9.5 (6.0-12.0)	12.0 (7.0-13.0)	6.0 (6.0-12.0)	.10
Group disparities in health care (3-15)	12.0 (12.0-13.0)	12.0 (8.0-12.5)	12.0 (10.5-15.0)	.43
Lack of support from health care providers (3-15)	7.5 (7.0-8.0)	8.0 (7.0-8.5)	7.0 (6.5-8.0)	.14
Diabetes stigma				
Treated differently (6-30)	11.5 (10.0-12.0)	12.0 (12.0-14.0)	6.5 (6.0-13.5)	.13
Blame and judgment (7-35)	23.0 (18.0-27.0)	20.5 (16.0-26.0)	18.0 (13.5-28.0)	.71
Self-stigma (6-30)	10.5 (10.0-12.0)	10.0 (9.5-12.0)	9.0 (5.0-10.0)	.07
Racial discrimination (0-9)	0.5 (0.0-1.0)	0.0 (0.0-1.5)	0.0 (0.0-0.0)	.30
Worry (0-12)	8.0 (7.0-9.0)	7.0 (7.0-8.5)	7.0 (7.0-8.0)	.59
Major discrimination (0-9)	1.5 (0.0-3.0)	1.0 (0.0-3.0)	1.0 (0.0-2.0)	.64
Everyday discrimination (0-30)	0.0 (0.0-1.0)	0.0 (0.0-1.5)	0.0 (0.0-1.0)	.55

When evaluating the results by stage of liver disease, 20% (10/50) of the survey participants had no cirrhosis, 56% (28/50) had compensated cirrhosis, and 24% (12/50) had decompensated cirrhosis. Respondents with no cirrhosis were older, mostly female (7/10, 70%), 20% (2/10) Hispanic individuals, 80% (8/10) White individuals, and more likely to be insured through their employers. In comparison, respondents with compensated cirrhosis were younger, on Medicaid and Medicare, and more racially and ethnically diverse (1/28, 3.6% Black individuals; 4/28, 14.3% Hispanic individuals; 21/28, 75% White individuals; and 2/28, 7.1% other [not Hispanic]). Participants with compensated cirrhosis also encompassed the highest proportion of respondents with an annual household income of less than US $35,000. Participants with decompensated cirrhosis were aged 63.8 (SD 9.74) years on average, 16.7% (2/12) employed, White individuals (12/12, 100%), and more likely to have private health insurance.

### Laboratory Results and Liver Disease Progression

Table 3 shows laboratory results and relevant medical history. Black and Hispanic participants appeared to have less advanced liver disease as 28.6% (2/7) had no cirrhosis and 71.4% (5/7) had compensated cirrhosis, whereas 53.5% (23/43) and 27.9% (12/43) of their non-Black and non-Hispanic counterparts had compensated and decompensated cirrhosis, respectively. White participants also had higher rates of comorbidities such as hypertension (32/43, 74.4%) and chronic kidney disease (9/43, 20.9%), but Black and Hispanic participants had higher prevalence of heart disease (2/7, 28.6%), hyperlipidemia (6/7, 85.7%), and neuropathy or diabetic retinopathy (3/7, 42.9%). In general, White participants had more elevated laboratory results in blood sugar and liver enzyme measures, including hemoglobin A_1c_ (median 6.5%, IQR 5.9%-7.4%), alanine transaminase (median 38.0, IQR 27.0-46.0 U/L), aspartate transaminase (median 35.0, IQR 26.0-47.0 U/L), alkaline phosphatase (median 96.0, IQR 76.0-128.0 U/L), and prothrombin time (median 11.5, IQR 10.5-12.4 seconds). In contrast, the Black and Hispanic group had higher albumin levels (median 4.5, IQR 4.4-4.6 g/dL). The differences in direct bilirubin and international normalized ratio were negligible. Regarding diabetes medication, metformin was most popular across the board, followed by long-acting insulin and semaglutide injections.

**Table 3.** Medical history pertaining to metabolic dysfunction–associated steatotic liver disease progression.

	Black and Hispanic participants (n=7)	Not Black or Hispanic participants (n=43)	*P* value
Stage of liver disease, n (%)			.27
No cirrhosis	2 (28.6)	8 (18.6)	
Compensated cirrhosis	5 (71.4)	23 (53.5)	
Decompensated cirrhosis	0 (0)	12 (27.9)	
Common comorbidities, n (%)			
Heart disease	2 (28.6)	6 (14.0)	.47
Hypertension	5 (71.4)	32 (74.4)	.30
Hyperlipidemia	6 (85.7)	23 (53.5)	.01
Chronic kidney disease	1 (14.3)	9 (20.9)	>.99
Neuropathy and retinopathy	3 (42.9)	9 (20.9)	.13
Hemoglobin A_1c_ (%), median (IQR)	6.0 (5.7-6.4)	6.5 (5.9-7.4)	.15
ALT^a^ (U/L), median (IQR)	31.0 (25.0-47.0)	38.0 (27.0-46.0)	.81
AST^b^ (U/L), median (IQR)	32.0 (25.0-38.0)	35.0 (26.0-47.0)	.22
Albumin (g/dL), median (IQR)	4.5 (4.4-4.6)	4.1 (3.7-4.5)	.04
Direct bilirubin (mg/dL), median (IQR)	0.2 (0.2-0.4)	0.3 (0.2-0.5)	.47
Alkaline phosphatase (U/L), median (IQR)	82.0 (66.0-113.0)	96.0 (76.0-128.0)	.55
PT^c^ (s), median (IQR)	11.1 (10.5-11.8)	11.5 (10.5-12.4)	.46
INR^d^, median (IQR)	1.1 (1.0-1.1)	1.1 (1.0-1.1)	.86
Diabetes medication, n (%)			
Metformin	4 (57.1)	19 (44.2)	.15
Empagliflozin	1 (14.3)	7 (16.3)	.80
Semaglutide injection	2 (28.6)	7 (16.3)	.19
Tirzepatide	1 (14.3)	9 (20.9)	>.99
Long-acting insulin	2 (28.6)	13 (30.2)	.63

a ALT: alanine transaminase.

b AST: aspartate transaminase.

c PT: prothrombin time.

d INR: international normalized ratio.

### SDOH Results

The median health literacy, medical mistrust, diabetes stigma, and racism and discrimination scores classified by race and ethnicity are shown in Table 4. In general, Black and Hispanic patients had a stronger sense of medical mistrust, significantly in the “lack of support from health care providers” category, and they experienced more suspicion and group disparities in health care. Black and Hispanic patients also experienced more diabetes stigma for subcategories such as “Blame and judgement” and “Self-stigma.” Major instances of discrimination, which entail discriminatory experiences attributable to factors outside of race such as sex, weight, and educational level, varied slightly by race and ethnicity, but Black and Hispanic individuals did report significantly more experiences of racial discrimination and worry about being discriminated against. No differences were noted in health literacy and everyday discrimination among races and ethnicities. Let it be noted that, due to the broad categorization offered by the Rapid Estimate of Adult Literacy in Medicine–Short Form test, a ceiling effect was observed in all health literacy results. More details on the distribution of health literacy scores can be found in Multimedia Appendix 4.

**Table 4.** Social determinant of health median scores divided by race and ethnicity.

	Black and Hispanic participants (n=7), median (IQR)	Not Black or Hispanic participants (n=43), median (IQR)	*P* value	Hodges-Lehmann median difference
Health literacy (0-7)	7.0 (7.0 to 7.0)	7.0 (7.0 to 7.0)	.15	0.0 (0.0 to 4.0)
Group-based medical mistrust				
Suspicion (6-30)	14.0 (6.0 to 18.0)	12.0 (6.0 to 12.0)	.12	2.0 (0.0 to 6.0)
Group disparities in health care (3-15)	8.0 (6.0 to 12.0)	12.0 (11.0 to 15.0)	.06	−3.0 (–6.0 to 0.0)
Lack of support from health care providers (3-15)	8.0 (8.0 to 10.0)	7.0 (7.0 to 8.0)	.03	1.0 (0.0 to 3.0)
Diabetes stigma				
Treated differently (6-30)	11.0 (6.0 to 14.0)	12.0 (7.0 to 14.0)	.73	0.0 (–5.0 to 2.0)
Blame and judgment (7-35)	22.0 (19.0 to 28.0)	20.0 (14.0 to 26.0)	.47	2.0 (–4.0 to 7.0)
Self-stigma (6-30)	12.0 (5.0 to 14.0)	10.0 (8.0 to 12.0)	.56	0.0 (–4.0 to 4.0)
Racial discrimination (0-9)	3.0 (0.0 to 6.0)	0.0 (0.0 to 1.0)	.01	3.0 (0.0 to 5.0)
Worry (0-12)	10.0 (9.0 to 11.0)	7.0 (7.0 to 8.0)	.005	2.0 (1.0 to 3.0)
Major discrimination (0-9)	2.0 (0.0 to 3.0)	1.0 (0.0 to 3.0)	.38	0.0 (–1.0 to 2.0)
Everyday discrimination (0-30)	0.0 (0.0 to 3.0)	0.0 (0.0 to 1.0)	.39	0.0 (0.0 to 2.0)

The median health literacy, medical mistrust, diabetes stigma, and racism and discrimination scores classified by stage of liver disease are shown in Table 2. Patients with no cirrhosis had higher diabetes stigma, worry, and major and racial discrimination scores. Meanwhile, patients with compensated cirrhosis had higher scores for group-based medical mistrust. However, patients with decompensated cirrhosis were the least suspicious of health care providers and systems and felt most supported by providers. They were also the least concerned about being treated differently and feeling blame or judgment for having diabetes. Overall, survey participants with decompensated cirrhosis were observed to have fewer negative experiences with SDOH. Much like the race and ethnicity analysis, no differences were noted in health literacy, everyday discrimination, and group disparities in health care.

## Discussion

### Principal Findings

We explored health outcomes and SDOH divided by race and ethnicity and severity of liver disease. Black and Hispanic respondents were more likely to report an annual household income of less than US $35,000, use Medicaid and/or Medicare insurance, and experience racial discrimination and diabetes stigma. They also had more comorbidities, including heart disease, hyperlipidemia, and neuropathy and diabetic retinopathy. Cirrhosis groups tended to have lower incomes and public health insurance.

Our observations of greater medical mistrust among Black and Hispanic patients, especially in lack of support from health care providers (*P*=.03), align with findings from numerous prior studies [22-24] where patients reported mistrust across individual physicians, health systems, and modern medicine in general. This mistrust is likely rooted in both historical and personal maltreatment from health care institutions and can result in the nondisclosure of symptoms and conditions that are highly stigmatized, such as T2DM [25]. Patients with T2DM have a greater chance of being characterized as noncompliant by health care providers [26]. Stigmatizing attitudes among providers are also associated with less patient-centered treatment plans and lower confidence in provision of care [26]. Such stigma not only undermines patient-provider relationships but also perpetuates health disparities and worsens diabetes management. In our study, Black and Hispanic patients were found to have higher rates of diabetes self-stigma and fear of judgment from peers and providers. Increasing patient education and encouraging providers to reflect on their biases during training can help address stigma and potential miscommunication [25].

Limited health literacy represents a significant obstacle to treatment engagement as a patient’s ability to comprehend diabetes and liver disease–related information is hindered [27]. It can also contribute to negative experiences when accessing care, such as medical mistrust, and result in nonadherence, thus ultimately affecting outcomes and prognosis [28]. Prior liver disease and diabetes research [28,29] has documented lower health literacy among Black and Hispanic patients, but such results cannot be ascertained from our study. There may be a potential bias in the ability of highly educated patients to navigate referrals and insurance approvals to access tertiary care centers such as Michigan Medicine. In a study from the Institute for Clinical Evaluative Sciences, adults from neighborhoods with higher educational levels in Ontario were 30% more likely to be referred to a specialist, which, in that case, was a psychiatrist [30]. Additionally, because of the ceiling effect demonstrated by the sample cohort responses, health literacy scores must be interpreted with caution, and alternative or complementary health literacy measures may be required for a larger future study.

When individual SDOH distribution was evaluated in patients without cirrhosis, with compensated cirrhosis, or with decompensated cirrhosis, lower income was more common in cirrhosis groups. Our results align with the conclusions drawn from our preliminary study, where higher disadvantage scores—defined as households led by a single mother, receiving public assistance income, under the poverty level, or with no employed member—were linked to higher rates of hepatic decompensation [7]. The cirrhosis incidence in lower-income patients, combined with the growing total hospitalization costs for patients with compensated and decompensated cirrhosis [31], highlights the need to reduce the financial burden of specialty care. Additionally, Medicare and Medicaid use increased with cirrhosis severity, and the number of employer-provided health insurance holders was significantly lower among those with cirrhosis (*P*=.04). Patients on public health insurance have shown to have lower rates of viral hepatitis and hepatocellular carcinoma screening [32,33], which is concerning as MASLD is a leading cause of hepatocellular carcinoma.

A key strength of this study is its comprehensive SDOH assessment—which was directly linked to medical chart data from actively managed patients—thus allowing for a more accurate and clinically relevant analysis. However, several limitations must be acknowledged. As this study was conducted at a large tertiary specialty care center, there is potential for referral bias. Most participants (28/50, 56%) had compensated cirrhosis, likely due to being referred to the transplant clinic, which may limit the generalizability of the findings to broader cirrhotic populations. Additionally, the cross-sectional design of this study restricts our ability to infer causal relationships, so we are only able to note observed associations. Furthermore, the survey design may have impacted measurement accuracy. Because the survey was administered by research staff and many of the measures comprised self-reported single items, concerns about social desirability may have influenced participant answers to sensitive topics. The use of a convenience sample also limits the representativeness of the study population. We aimed to recruit at least 4 Hispanic patients and 7 Black patients. Although we succeeded in oversampling Hispanic patients, we failed to reach our target for Black patients despite extending recruitment to a partnering Michigan Medicine hospital that serves a more diverse pool of patients.

Between races and ethnicities, Black and Hispanic respondents reported significantly more instances of racial discrimination (*P*=.01) and lifetime worries about being discriminated against (*P*=.005). However, it is important to acknowledge that our survey derived its racial categories from the US Office of Management and Budget guidelines that are used by the US census, which specifies 5 major racial groups—White or Caucasian, Black or African American, American Indian or Alaska Native, Asian, and Native Hawaiian or other Pacific Islander—and 2 ethnicities—Hispanic or not Hispanic. Therefore, the discrimination and medical mistrust scores and SDs, as well as comorbidity distribution, for White patients may be inflated as people from areas such as the Middle East and North Africa (MENA) are considered to be “White.” This racial aggregation also masks health and SDOH disparities that exist in Arab or MENA Americans, who have a higher prevalence of cardiovascular and metabolic diseases than White individuals of European descent [34]. In this study, 4.7% (2/43) of the White, non-Hispanic participants identified as being from the MENA region, and they reported experiencing more discrimination and mistrust.

### Conclusions

This exploratory, cross-sectional study linked survey and medical chart data to investigate the distribution of individual-level SDOH in patients with T2DM and MASLD. Black and Hispanic patients reported significantly more instances of racial discrimination and medical mistrust, along with lower incomes and public health insurance coverage. In this small cross-sectional sample, no clear descriptive pattern in these SDOH measures was observed across liver disease stage. These hypothesis-generating findings will help inform a future larger study, where we will assess and develop interventions to address the combined impact of individual- and neighborhood-level SDOH on health-related longitudinal outcomes in patients with MASLD and T2DM.

## Supplementary material

10.2196/91608 Multimedia Appendix 1 Components of individual-level social determinant of health measurements.

10.2196/91608 Multimedia Appendix 2 Sociodemographic characteristics and social determinant of health median scores with alternative racial and ethnic groupings.

10.2196/91608 Multimedia Appendix 3 Income breakdown by race and ethnicity.

10.2196/91608 Multimedia Appendix 4 Distribution of health literacy scores.

## Abbreviations

EHR: electronic health record
MASLD: metabolic dysfunction–associated steatotic liver disease
MENA: Middle East and North Africa
SDOH: social determinants of health
STROBE: Strengthening the Reporting of Observational Studies in Epidemiology
T2DM: type 2 diabetes mellitus
