# Supplementary material for: Characterizing Social Determinants of Health in Patients With Type 2 Diabetes and Liver Disease: Cross-Sectional Survey Study
Source: JMIR Form Res. 2026 Jun 15;10:e91608. doi: 10.2196/91608 (PMC13268636; doi:10.2196/91608)
Supplement: Multimedia Appendix 1 [file formative-v10-e91608-s001.docx]

**Supplement 1: Components of Individual-level SDOH Measurements**

| Factor | Survey name/source | Reliability |
| --- | --- | --- |
| Income | Ask for household income and size | N/A |
| Education | Ask for the maximum level of education completed | N/A |
| Health literacy | REALM-SF^1^ | r = 0.94 |
| English proficiency | United States Census Bureau^2^ | N/A |
| Health-related stigma | DSAS-2^3^ | r = 0.78, p < 0.001 |
| Racism | CARDIA study^4^ | N/A |
| Health insurance | Review of medical record | N/A |
| Alcohol intake | AUDIT-C^5^ | r = 0.91 |
| Tobacco use | Global Tobacco Surveillance System^6^ (subset) | N/A |
| Injection drug use | Ask about history of injection drug use | N/A |
| Trust in healthcare | Group Based Medical Mistrust^7-9^ | α = 0.87 |

1. Arozullah AM, Yarnold PR, Bennett CL, et al. Development and validation of a short-form, rapid estimate of adult literacy in medicine. *Med Care*. Nov 2007;45(11):1026-33. doi:10.1097/MLR.0b013e3180616c1b

2. USCBAC. Why We Ask Each Question. *Survey*. Vol 2023

3. Browne JL, Ventura AD, Mosely K, Speight J. Measuring the Stigma Surrounding Type 2 Diabetes: Development and Validation of the Type 2 Diabetes Stigma Assessment Scale (DSAS-2). *Diabetes Care*. Dec 2016;39(12):2141-2148. doi:10.2337/dc16-0117

4. Borrell LN, Kiefe CI, Diez-Roux AV, Williams DR, Gordon-Larsen P. Racial discrimination, racial/ethnic segregation, and health behaviors in the CARDIA study. *Ethn Health*. 2013;18(3):227-43. doi:10.1080/13557858.2012.713092

5. Bush K, Kivlahan DR, McDonell MB, Fihn SD, Bradley KA. The AUDIT alcohol consumption questions (AUDIT-C): an effective brief screening test for problem drinking. Ambulatory Care Quality Improvement Project (ACQUIP). Alcohol Use Disorders Identification Test. *Arch Intern Med*. Sep 14 1998;158(16):1789-95. doi:10.1001/archinte.158.16.1789

6. Global Tobacco Surveillance System Collaborating G. Global Tobacco Surveillance System (GTSS): purpose, production, and potential. *J Sch Health*. Jan 2005;75(1):15-24. doi:10.1111/j.1746-1561.2005.tb00004.x

7. Thompson HS, Valdimarsdottir HB, Winkel G, Jandorf L, Redd W. The Group-Based Medical Mistrust Scale: psychometric properties and association with breast cancer screening. *Prev Med*. Feb 2004;38(2):209-18. doi:10.1016/j.ypmed.2003.09.041

8. Shelton RC, Winkel G, Davis SN, et al. Validation of the group-based medical mistrust scale among urban black men. *J Gen Intern Med*. Jun 2010;25(6):549-55. doi:10.1007/s11606-010-1288-y

9. Martinez B, Huh J, Tsui J. Validating the Group-Based Medical Mistrust Scale with English and Spanish Speaking Latino Parents of Adolescents. *J Am Board Fam Med*. Mar-Apr 2022;35(2):244-254. doi:10.3122/jabfm.2022.02.210307
