# Supplementary material for: Characterizing Social Determinants of Health in Patients With Type 2 Diabetes and Liver Disease: Cross-Sectional Survey Study
Source: JMIR Form Res. 2026 Jun 15;10:e91608. doi: 10.2196/91608 (PMC13268636; doi:10.2196/91608)
Supplement: Multimedia Appendix 2 [file formative-v10-e91608-s002.docx]

**Supplement 2a: Sociodemographic Characteristics and SDOH Median Scores, Divided by Race/Ethnicity**

|  | Hispanic (n=6) | Not Hispanic* (n=44) | p-value | Hodges Lehmann |
| --- | --- | --- | --- | --- |
| Demographics, n (%): |  |  |  |  |
| Age [SD] | 65 [±9.0] | 62 [±9.0] | 0.53 | 2.0 (-5.0 to 11.0) |
| Sex, female | 2 (33) | 27 (61) | 1.00 |  |
| Employed | 2 (33) | 15 (34) | 0.59 |  |
| Household income: |  |  | 0.60 |  |
| <$35,000 | 3 (50) | 9 (20) |  |  |
| $35,000 to $49,999 | 0 (0) | 8 (18) |  |  |
| $50,000 to $74,999 | 1 (17) | 6 (14) |  |  |
| $75,000 to $99,999 | 1 (17) | 3 (7) |  |  |
| >$100,000 | 1 (17) | 13 (30) |  |  |
| Education: |  |  | 0.36 |  |
| Less than high school graduation | 0 (0) | 1 (2) |  |  |
| High school graduation | 0 (0) | 9 (21) |  |  |
| College | 3 (50) | 29 (65) |  |  |
| Graduate degree | 3 (50) | 5 (12) |  |  |
| Insurance: |  |  | 0.68 |  |
| Medicaid | 2 (33) | 7 (16) |  |  |
| Medicare | 2 (33) | 15 (34) |  |  |
| Marketplace | 0 (0) | 2 (5) |  |  |
| Private (employer) | 2 (33) | 20 (45) |  |  |
| SDOH scores [IQR]: |  |  |  |  |
| Health literacy | 7.0 [7.0 to 7.0] | 7.0 [0.0 to 7.0] | 0.21 | 0.0 (0.0 to 4.0) |
| Group-based medical mistrust: |  |  |  |  |
| Suspicion | 13.0 [6.0 to 18.0] | 12.0 [6.0 to 12.0] | 0.27 | 2.0 (-1.0 to 6.0) |
| Group disparities in healthcare | 9.0 [6.0 to 12.0] | 12.0 [10.5 to 14.5] | 0.13 | -3.0 (-6.0 to 0.0) |
| Lack of support from healthcare providers | 9.0 [8.0 to 10.0] | 7.5 [7.0 to 8.0] | 0.03 | 1.0 (0.0 to 3.0) |
| Diabetes stigma: |  |  |  |  |
| Treated differently | 10.5 [6.0 to 14.0] | 12.0 [8.5 to 14.0] | 0.43 | -1.0 (-6.0 to 2.0) |
| Blame and judgement | 23.0 [19.0 to 28.0] | 20.0 [14.5 to 26.0] | 0.41 | 2.0 (-4.0 to 8.0) |
| Self-stigma | 12.0 [5.0 to 14.0] | 10.0 [8.5 to 12.0] | 0.48 | 2.0 (-5.0 to 4.0) |
| Racial discrimination | 2.0 [0.0 to 5.0] | 0.0 [0.0 to 1.0] | 0.05 | 1.0 (0.0 to 4.0) |
| Worry | 9.5 [9.0 to 10.0] | 7.0 [7.0 to 8.0] | 0.03 | 2.0 (1.0 to 3.0) |
| Major discrimination | 1.5 [0.0 to 3.0] | 1.0 [0.0 to 3.0] | 0.66 | 0.0 (-1.0 to 2.0) |
| Everyday discrimination | 0.0 [0.0 to 2.0] | 0.0 [0.0 to 1.0] | 0.94 | 0.0 (0.0 to 1.0) |
| *Black respondent is included in ‘Not Hispanic’ | | | | |

**Supplement 2b: Sociodemographic Characteristics and SDOH Median Scores, Divided by Race/Ethnicity****

|  | Hispanic (n=6) | Not Hispanic, White (n=43) | p-value | Hodges Lehmann |
| --- | --- | --- | --- | --- |
| Demographics, n (%): |  |  |  |  |
| Age [SD] | 65 [±9.0] | 63 [±9.1] | 0.56 | 2.0 (-5.0 to 11.0) |
| Sex, female | 2 (33) | 26 (60) | 0.38 |  |
| Employed | 2 (33) | 14 (3) | 1.00 |  |
| Household income: |  |  | 0.59 |  |
| <$35,000 | 3 (50) | 9 (21) |  |  |
| $35,000 to $49,999 | 0 (0) | 8 (19) |  |  |
| $50,000 to $74,999 | 1 (17) | 5 (12) |  |  |
| $75,000 to $99,999 | 1 (17) | 3 (7) |  |  |
| >$100,000 | 1 (17) | 13 (30) |  |  |
| Education: |  |  | 0.38 |  |
| Less than high school graduation | 0 (0) | 1 (2) |  |  |
| High school graduation | 0 (0) | 9 (21) |  |  |
| College | 3 (50) | 28 (65) |  |  |
| Graduate degree | 3 (50) | 5 (12) |  |  |
| Insurance: |  |  | 0.68 |  |
| Medicaid | 2 (33) | 7 (16) |  |  |
| Medicare | 2 (33) | 14 (33) |  |  |
| Marketplace | 0 (0) | 2 (5) |  |  |
| Private (employer) | 2 (33) | 20 (45) |  |  |
| SDOH scores [IQR]: |  |  |  |  |
| Health literacy | 7.0 [7.0 to 7.0] | 7.0 [0.0 to 7.0] | 0.20 | 0.0 (0.0 to 4.0) |
| Group-based medical mistrust: |  |  |  |  |
| Suspicion | 13.0 [6.0 to 18.0] | 12.0 [6.0 to 12.0] | 0.25 | 2.0 (-1.0 to 6.0) |
| Group disparities in healthcare | 9.0 [6.0 to 12.0] | 12.0 [11.0 to 15.0] | 0.12 | -3.0 (-6.0 to 0.0) |
| Lack of support from healthcare providers | 9.0 [8.0 to 10.0] | 7.5 [7.0 to 8.0] | 0.03 | 1.0 (0.0 to 3.0) |
| Diabetes stigma: |  |  |  |  |
| Treated differently | 10.5 [6.0 to 14.0] | 12.0 [7.0 to 14.0] | 0.45 | -1.0 (-6.0 to 3.0) |
| Blame and judgement | 23.0 [19.0 to 28.0] | 20.0 [14.5 to 26.0] | 0.42 | 2.0 (-4.0 to 8.0) |
| Self-stigma | 12.0 [5.0 to 14.0] | 10.0 [8.0 to 12.0] | 0.49 | 2.0 (-5.0 to 5.0) |
| Racial discrimination | 2.0 [0.0 to 5.0] | 0.0 [0.0 to 1.0] | 0.03 | 1.0 (0.0 to 4.0) |
| Worry | 9.5 [9.0 to 10.0] | 7.0 [7.0 to 8.0] | 0.02 | 2.0 (1.0 to 3.0) |
| Major discrimination | 1.5 [0.0 to 3.0] | 1.0 [0.0 to 3.0] | 0.62 | 0.0 (-1.0 to 2.0) |
| Everyday discrimination | 0.0 [0.0 to 2.0] | 0.0 [0.0 to 1.0] | 0.86 | 0.0 (0.0 to 1.0) |
| **Black respondent is not included in this table | | | | |
