# Supplementary material for: Characterizing Social Determinants of Health in Patients With Type 2 Diabetes and Liver Disease: Cross-Sectional Survey Study
Source: JMIR Form Res. 2026 Jun 15;10:e91608. doi: 10.2196/91608 (PMC13268636; doi:10.2196/91608)
Supplement: Multimedia Appendix 3 [file formative-v10-e91608-s003.docx]

**Supplement 3: Race-Income Breakdown**

| Race/ethnicity | Income |
| --- | --- |
| Black (n=1) | $50,000-74,999 |
| Hispanic (n=6) | Less than $35,000 (3)  $50,000-74,999 (1)  $75,000-99,999 (1)  $100,000 or more (1) |
| White, not Hispanic (n=42) | Less than $35,000 (9)  $35,000-49,999 (8)  $50,000-74,999 (5)  $75,000-99,999 (3)  $100,000 or more (12)  Prefer not to say/IDK (5) |
| Other, not Hispanic (n=1) | $100,000 or more |
