# Supplementary figures and images for: Characterizing Social Determinants of Health in Patients With Type 2 Diabetes and Liver Disease: Cross-Sectional Survey Study
Source: JMIR Form Res. 2026 Jun 15;10:e91608. doi: 10.2196/91608 (PMC13268636; doi:10.2196/91608)

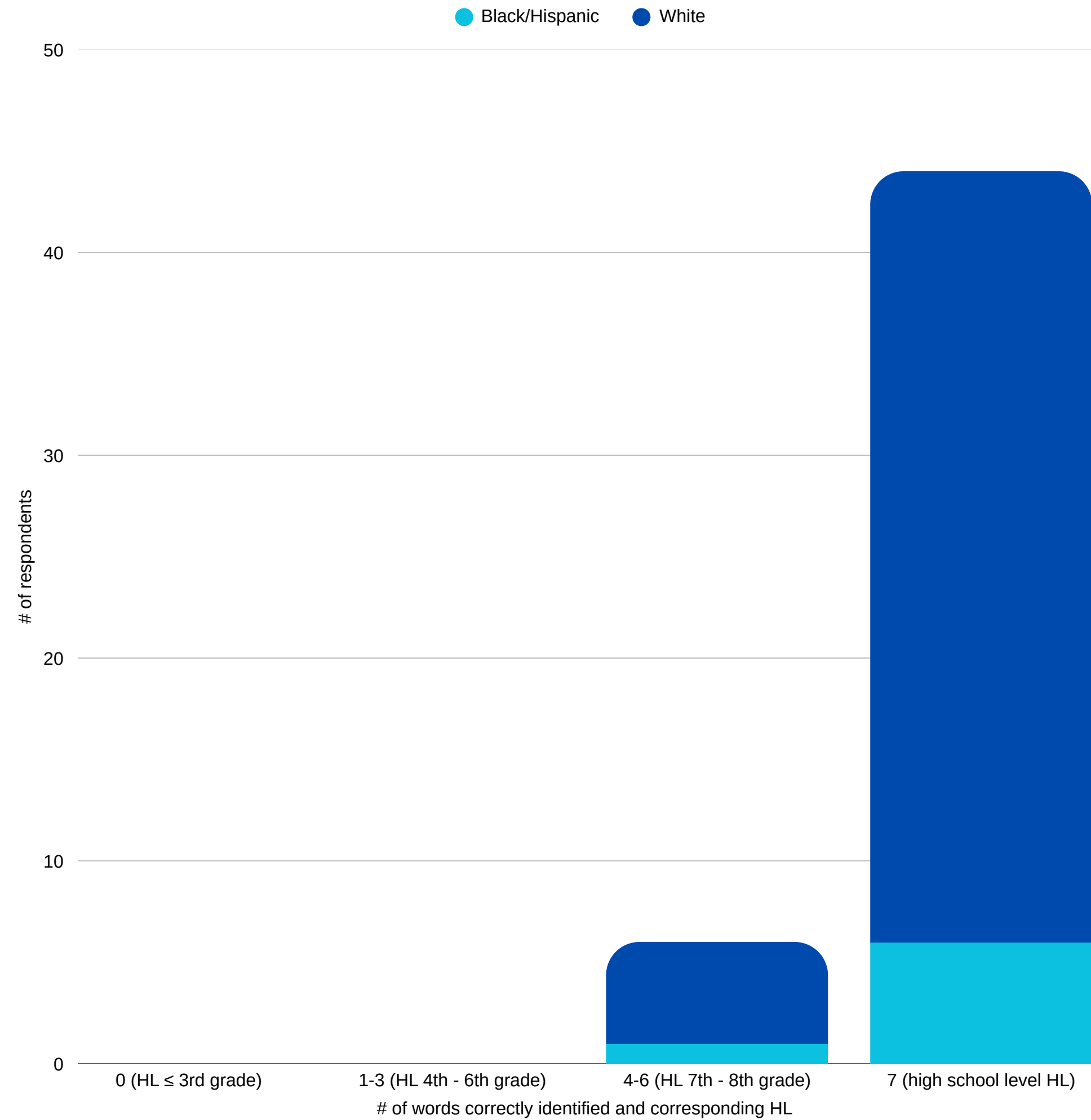

Supplement: Multimedia Appendix 4 [file formative-v10-e91608-s004.pdf]
